# Social Behavior, Community Composition, Pathogen Strain, and Host Symbionts Influence Fungal Disease Dynamics in Salamanders

**DOI:** 10.3389/fvets.2021.742288

**Published:** 2021-11-29

**Authors:** Mae Cowgill, Andrew G. Zink, Wesley Sparagon, Tiffany A. Yap, Hasan Sulaeman, Michelle S. Koo, Vance T. Vredenburg

**Affiliations:** ^1^Department of Biology, San Francisco State University, San Francisco, CA, United States; ^2^Daniel K. Inouye Center for Microbial Oceanography: Research and Education, Department of Oceanography and Sea Grant College Program, UUniversity of Hawai‘i at Mānoa, HI, United States; ^3^Center for Biological Diversity, Oakland, CA, United States; ^4^Museum of Vertebrate Zoology, University of California, Berkeley, Berkeley, CA, United States

**Keywords:** chytridiomycosis, sociality, symbiotic bacteria, historical prevalence, microbiome, *Batrachochytrium dendrobatidis*, *Aneides lugubris*, *Batrachoseps luciae*

## Abstract

The emerging fungal pathogen, *Batrachochytrium dendrobatidis* (*Bd*), which can cause a fatal disease called chytridiomycosis, is implicated in the collapse of hundreds of host amphibian species. We describe chytridiomycosis dynamics in two co-occurring terrestrial salamander species, the Santa Lucia Mountains slender salamander, *Batrachoseps luciae*, and the arboreal salamander, *Aneides lugubris*. We (1) conduct a retrospective *Bd*-infection survey of specimens collected over the last century, (2) estimate present-day *Bd* infections in wild populations, (3) use generalized linear models (GLM) to identify biotic and abiotic correlates of infection risk, (4) investigate susceptibility of hosts exposed to *Bd* in laboratory trials, and (5) examine the ability of host skin bacteria to inhibit *Bd* in culture. Our historical survey of 2,866 specimens revealed that for most of the early 20th century (~1920–1969), *Bd* was not detected in either species. By the 1990s the proportion of infected specimens was 29 and 17% (*B. luciae* and *A. lugubris*, respectively), and in the 2010s it was 10 and 17%. This was similar to the number of infected samples from contemporary populations (2014–2015) at 10 and 18%. We found that both hosts experience signs of chytridiomycosis and suffered high *Bd*-caused mortality (88 and 71% for *B. luciae* and *A. lugubris*, respectively). Our GLM revealed that *Bd*-infection probability was positively correlated with intraspecific group size and proximity to heterospecifics but not to abiotic factors such as precipitation, minimum temperature, maximum temperature, mean temperature, and elevation, or to the size of the hosts. Finally, we found that both host species contain symbiotic skin-bacteria that inhibit growth of *Bd* in laboratory trials. Our results provide new evidence consistent with other studies showing a relatively recent *Bd* invasion of amphibian host populations in western North America and suggest that the spread of the pathogen may be enabled both through conspecific and heterospecific host interactions. Our results suggest that wildlife disease studies should assess host-pathogen dynamics that consider the interactions and effects of multiple hosts, as well as the historical context of pathogen invasion, establishment, and epizootic to enzootic transitions to better understand and predict disease dynamics.

## Introduction

Amphibians are an ancient and diverse lineage of vertebrates [~370 mya; currently 8,345 species; (1, 2)] that have survived the last four global mass extinction events (3–5). Their biphasic lifestyle links aquatic and terrestrial productivity (6), and they fill key positions in food webs because of their large population sizes and the fact that they serve as abundant consumers, and as prey to other species (7). However, amphibians have recently experienced extinctions and severe global population declines due to a combination of factors, including habitat loss and fragmentation, environmental contaminants, disease, and climate change (3, 5, 8, 9).

An emerging infectious fungal disease, chytridiomycosis, has captured the attention of biologists and the general public because it has driven die-offs in hundreds of amphibian species around the world (3, 9–13). This disease is caused by the chytridiomycete fungi, *Batrachochytrium dendrobatidis* (*Bd*) (14) and *Batrachochytrium salamandrivorans* (*Bsal*) (15), which infect and disrupt skin function, including osmoregulation, and can lead to death (15–18). While less is known about *Bsal*, the impact of the *Bd* pathogen on amphibians represents the worst known case of disease among vertebrates in recorded history (9, 12). To predict the effects that *Bd* chytridiomycosis will continue to have on host species, it is essential to understand longitudinal patterns of *Bd* infection prevalence [e.g., temporal pattern of pathogen invasion, epizootic outbreak, and either establishment or loss; (19, 20)], host species susceptibility patterns (influenced by skin microbiomes, social behavior and interactions with heterospecifics), and host-*Bd* dynamics so that models can be used to understand and predict the interactions across a wide range of circumstances (20).

Though *Bd* chytridiomycosis has mostly been studied in anurans (Order Anura: frogs and toads), salamanders and newts (Order Caudata) are also known to be affected by *Bd* (12, 21–25). In the North America salamander biodiversity hotspot which contains ~50% of all known species (1, 26), very little is known about which salamander species have declined and if *Bd* is the cause of such declines (12). The west coast of North America has the second most diverse assemblage of salamander species in North America (after Appalachia region), and 64% (49/77) of its extant amphibian species are salamanders (1). Whereas, the emergence of *Bd* is closely linked to mass die-offs and local extinctions in anurans in California (17, 19, 27, 28), the relationship between *Bd* emergence and population declines is not well-characterized in salamanders (21, 22, 25). Lack of attention to salamander disease dynamics in California may be attributed to the fact that most salamander species are fossorial or semi-fossorial (living under leaf litter and woody debris), nocturnal, and solitary breeders; as such, many abundance estimates suffer from ascertainment bias (29). Furthermore, in California, a lack of consistent historical population sampling [relative to anurans; (27)] makes it difficult to confidently assess whether salamander populations have declined.

In California, recent studies have shown a consistent pattern of no or almost no *Bd* early in the 20th century, followed by *Bd* invasion and emergence in amphibians in the late 1960s and 1970s (19, 21, 22, 25, 28, 30–32). The effects of this pathogen on salamanders, which in California represent a majority of the diversity of amphibians, are poorly understood. A better understanding of host pathogen dynamics requires contextual information such as spatial and temporal pathogen data (i.e., longitudinal pathogen data), host susceptibility, host behavior, immune responses, as well as other biotic and abiotic factors that may influence host survival (33–36). Another important factor is *Bd* lineage. Molecular studies reveal a complex evolutionary history in *Bd* (37), with significant differences in pathogenicity and in spatial patterns of spread between lineages [e.g., (12, 38–40)]. The pathogen is believed to have originated in Asia, and there are at least five major *Bd* lineages which vary in pathogenicity (12, 24, 38). The “global panzootic lineage” (*Bd-*GPL; used in this study) is associated with mass mortalities in wild amphibian populations (41), and to date is the only *Bd* lineage found in California (12, 39), but there is currently little or no information available on different populations of *Bd*, and their effects on hosts, in California.

**Figure 1:**
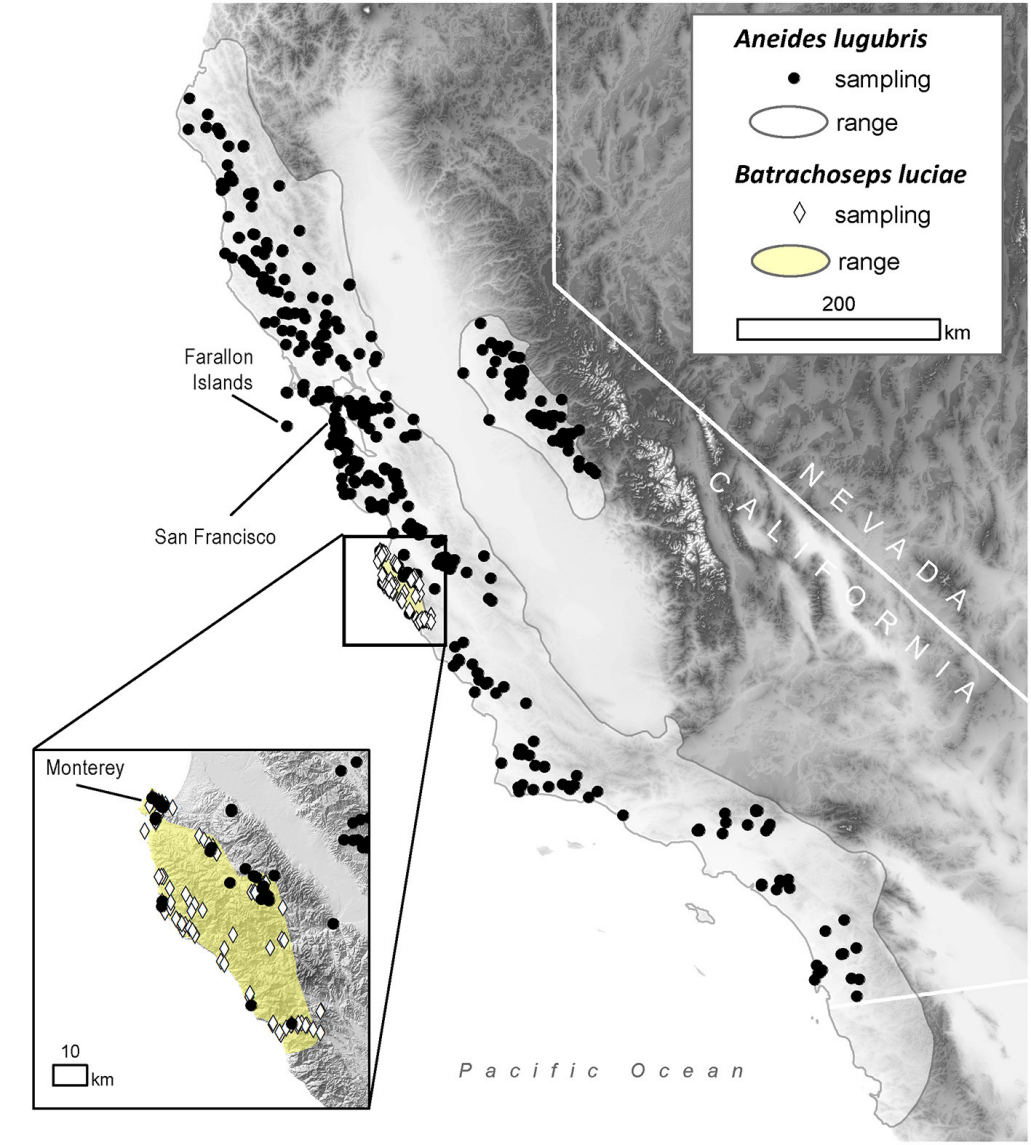
Distribution of sampling locations within the range of the arboreal salamander (*Aneides lugubris*; solid gray line) and the Santa Lucia Mountains slender salamander (*Batrachoseps luciae*; yellow area) in California, USA.

We investigate the proportion of individuals infected with the *Bd* pathogen in wild salamander populations and combine both field sampling and lab-based (i.e., experimental) studies to investigate pathogen host susceptibility and historical *Bd* occurrence in two species of co-occurring terrestrial salamanders: the Santa Lucia Mountains slender salamander (*Batrachoseps luciae*), and the arboreal salamander (*Aneides lugubris*). The arboreal salamander has a wide range that overlaps with much of the amphibian diversity in California, whereas this species of slender salamander has a very limited geographic range (42) (Figure 1), making them potentially more vulnerable to extinction (43, 44). In particular, in this study, we provide in-depth host/pathogen information on two sympatric terrestrial salamander host species: we (1) conduct a 90-year retrospective survey to document the proportion of *Bd*-infected hosts through time using specimens from natural history museum collections, (2) non-intrusively document the proportion of *Bd*-infected animals in contemporary wild populations, (3) use modeling to identify biotic (e.g., species overlap, group size) and abiotic (e.g., precipitation. temperature) infection correlates, (4) investigate host susceptibility in laboratory trials where hosts were exposed to either one or two populations of the *Bd* pathogen (one from a *Bd*-epizootic that led to frog population collapse, and another from focal hosts that were infected upon capture), and (5) examine the ability of naturally occurring amphibian host skin bacteria from our focal host species to inhibit *Bd* in culture.

We describe longitudinal patterns of *Bd*-infection in order to determine if they are consistent with previous studies on other *Bd* host species that occur in western North America, where several frog species have experienced *Bd* epizootics followed by mass die offs and population extinctions [e.g., *Rana muscosa, Rana sierrae*; (17)]. The prevailing hypothesis to explain disease dynamics in these species is that *Bd* invaded novel host populations causing epizootics, and surviving host populations eventually transitioned to more stable pathogen/host enzootic dynamics (19, 20, 28, 45). Theoretically, infection prevalence will peak during the epizootic and then decrease as the dynamics between the host and the pathogen shift toward an enzootic state (17, 20), but the effects of *Bd* on remaining populations could still be significant (46). Our approach combines a retrospective and contemporary view of host pathogen dynamics as we also test host *Bd*-infection susceptibility and look for correlates of *Bd* infection of individual hosts in wild populations. Finally, our skin bacterial cultures and *Bd* inhibition trials allow us to test whether these hosts might be protected from *Bd* infection by their skin microbiota. Thus, we use a combination of field and laboratory techniques to investigate the role of the *Bd* pathogen in populations of a widely occurring terrestrial salamander and a narrowly endemic terrestrial salamander in western North America, where *Bd* epizootics have severely affected other amphibian host species (3, 17, 19, 46, 47).

## Methods and Materials

### Historical Infection Study (1920–2015)

To estimate historical infection prevalence of *Bd* in populations of *B. luciae* and *A. lugubris*, we analyzed *Bd* occurrence in a total of 2,459 museum specimens (1,522 *B. luciae*, 936 *A. lugubris*) from natural history collections at the Museum of Vertebrate Zoology (MVZ), University of California Berkeley, and the California Academy of Sciences (CAS), San Francisco, California. For *B. luciae*, we aimed to sample all available specimens (1,522 specimens were sampled from MVZ; none were available at CAS). The salamander *A. lugubris* has a much wider distribution (Figure 1) and has many more available specimens (MVZ plus CAS = 3,882; VertNet.org); thus, to minimize costs, we used a stratified (by decade) random sampling design to select 936 specimens as a subsample. We randomly selected 40 specimens per decade (collection years include 1940–2015). If there were fewer than 40 available in a decade, we selected all available specimens for that decade. In addition, we also selected all available *A. lugubris* collected within the area of overlap with *B. luciae* (*n* = 78; Monterey County, California; Figure 1) to enable us to determine possible disease-related relationships between these co-occurring species.

In museum specimens, we used a standard skin swabbing technique to test for the presence of *Bd* (48–50). Specimens were stroked 30 times with a MW113 dry swab (Medical Wire and Equipment Company)-−10 times both dorsally and ventrally, and five times on each laterum, spanning the majority of individual's body length. To decrease the chance of cross contamination between specimens, each specimen was rinsed in 70% ethanol before swabbing and gloves were changed between handling every specimen (50). Swabs were stored in 1.5-mL microcentrifuge tubes at 4°C. We used standard *Bd* DNA extraction and real-time quantitative polymerase chain reaction methods to detect *Bd* from swabs collected [described in (48, 49)]. These methods have been validated both in live specimens and formalin-fixed museum specimens stored in 70% ethanol (50, 51). Considering the relatively small difference in *Bd* detection rate using qPCR for samples run in singlicate and triplicate (50), we ran a larger number of samples in singlicate rather than a smaller number of samples in triplicate to both minimize costs and increase the sampling power and the geographic and temporal spread of the samples. Genomic equivalent results were multiplied by 80, the dilution factor in qPCR sample preparation, to estimate the number of *Bd* zoospores on the entire swab [Zswab; *Bd*-infection intensity; (17, 20). Samples with a Zswab score > zero were defined as *Bd*-positive.

### Contemporary Field Study and Generalized Linear Model

Field surveys for contemporary disease prevalence were conducted in Monterey County, California, from May 2014 to March 2015 at the following locations: Don Dahvee Park (Monterey, CA), Veterans Memorial Park (Monterey, CA), Mission Trails Park (Carmel, CA), and Lynn “Rip” Van Winkle Open Space (Pacific Grove, CA). In this area, mixed pine and oak woodlands are dominant, and *B. luciae* and *A. lugubris* are found in close proximity to each other; often under the same cover objects (e.g., decomposing woody debris, downed logs, bark, sticks). *B. luciae* has a very limited distribution, occurring solely in the central coast region, primarily Monterey County (42), while *A. lugubris* has a wide distribution across coastal California and the Sierra Nevada foothills (52) (Figure 1). However, we focused our field sampling of *A. lugubris* to the sites where it is sympatric with *B. luciae*, but also collected outside the area of sympatry. Sites were surveyed by turning logs, rocks and other debris to locate salamanders beneath them.

All *B. luciae* and *A. lugubris* encountered were captured by hand and measured (snout-vent length; tail length, weight). From each individual, we collected one or two non-invasive skin swabs, in a method identical to those described above for museum specimens [but without an ethanol wash; (48); following (17)] before being released. If two skin swabs were taken, the first swab was used to culture skin bacterial symbionts (see “*Bd* inhibition” section below), the second skin swab was used to detect *Bd* using a qPCR Bd assay (48). If only one swab was taken, it was used to detect *Bd*. For each cover item where a salamander was found, the number of intra- and interspecific individuals found within 15 m of the focal individual were recorded. Individual animals were captured, sampled, and released at the site of capture within a 10-min period.

### Laboratory Host Susceptibility Trials

In addition to the catch-and-release animals, we collected 31 *B. luciae* and 18 *A. lugubris* and transported them alive to the laboratory for susceptibility trials. All *B. luciae* and 12 *A. lugubris* were collected in Monterey County, California, from July 2014 to March 2015. Severe drought made salamander fieldwork difficult, thus we expanded our collection locations for *A. lugubris* and collected 6 additional individuals outside of the range of *B. luciae* (from San Mateo and Calaveras counties, California) in January and March 2015. At the site of capture, each salamander was placed individually in a plastic container lined with moist paper towels and transported to the animal care facility at San Francisco State University, where they were kept in individual standard plastic mouse cages (19 × 29.2 × 2.7 cm) lined with moist paper towels for the duration of the experiment. Salamanders were fed twice a week and received 5–10 crickets or ~10 wingless *Drosophila* (*B. luciae* only) at each feeding. Clean cages were provided 1–2 times per week and temperature was maintained between 17 and 20°C. Each animal was checked visually every day and kept on a 12-h light and 12-h dark daily schedule.

Ten of the 31 *B. luciae* collected were found to be *Bd*-infected at the time of collection, verified using qPCR from field swabs. We called this experimental group “wild strain (WS) field-infected” and we followed the infection throughout the study period, testing weekly for *Bd* (as described below). All 10 of the WS field-infected animals were individually housed for the duration of the experiment. For the remaining uninfected *B. luciae* individuals collected from the field (*n* = 21), we split them into three experimental groups: two experimental inoculation groups using two different *Bd* strains (“GPL lab-inoculated” and “wild strain lab-inoculated”) and a sham-inoculated control group (“uninfected controls”). The first group included eight randomly chosen individuals (of the 21 uninfected) and was named “GPL lab-inoculated.” Here we used the global panzootic lineage, *Bd*-GPL [strain id # CJB57-(4)-p6; (39)], cultured from Southern Mountain yellow-legged frog (*Rana muscosa*) epizootics in the Sierra Nevada, California (17). Individuals were inoculated with 2 × 106 zoospores (confirmed via hemacytometer) suspended in 15 mL of sterile water. These salamanders were placed in petri dishes and exposed to the solution individually for 20 min per day for 5 consecutive days, using the protocol from a previous study (53).

The second experimental group involved eight randomly chosen *B. luciae* and was termed “wild strain (WS) lab-inoculated.” For the zoospore source, we placed a known wild-infected *A. lugubris* from the same population as the *B. luciae* in a small container with 30 mL of sterile water for 20 min allowing time for *Bd* zoospores to be shed into the water. This “*Bd* water” was then divided equally among eight 50-mL falcon tubes. Sterile water was added to total 5 mL of solution in each falcon tube. A single *B. luciae* individual was placed in each falcon tube with the liquid and left for 1 h. The process was repeated each day for a total of 10 consecutive days.

For the third experimental group, termed “uninfected controls,” the five remaining individuals received sham inoculations. For the uninfected control group, we followed the steps described for the second experimental group (WS lab-inoculated) with the only difference being that we used an uninfected *A. lugubris* (as determined by qPCR) instead of an infected *A. lugubris* to produce the sham “*Bd* water.” The process was repeated each day for 10 consecutive days, as described above.

For susceptibility trials with *A. lugubris*, we had fewer field-collected animals available. Therefore, we had fewer trials and did not use the *Bd-*GPL strain (i.e., there was no “GPL lab-inoculated” treatment). Of the 18 *A. lugubris* collected in the wild, 5 showed an initial infection and we labeled them “WS field-infected.” Of the 13 uninfected individuals, seven were inoculated using the same protocol as the WS lab-inoculated *B. luciae* experiment. The 30 mL of “*Bd* water” from soaking a known, wild-infected *A. lugubris* was evenly divided into 7 small plastic containers (150- mL volume); however, since these salamanders are much larger than *B. luciae*, we added more sterile water to total 30 mL in each container. This was repeated for 10 consecutive days. The remaining six uninfected *A. lugubris* were exposed to sham “*Bd* water” and kept as controls in the same manner as described above.

The initial post-inoculation swab (for all groups and both species including sham controls) was taken 48 h after the last exposure period, and swabs were subsequently taken weekly for 14 weeks. For all experiments, individuals were euthanized using MS-222 if they exhibited loss of righting reflex, leg-locking, lethargy, very high *Bd*-infection intensity [i.e., Vredenburg's 10,000 Zoospore Rule; (17, 54)], or other signs indicative of severe chytridiomycosis (55).

### Host Skin Bacteria *Bd* Inhibition Trials

Bacteria were collected from the skin of 48 salamanders (40 *B. luciae* and 8 *A. lugubris*) and one clutch of *A. lugubris* eggs in the field. All individuals in this trial were captured in Don Dahvee Park, Monterey, CA and were handled with sterile nitrile gloves and sterile plastic bags. Animals were rinsed thoroughly with 50 mL of sterile water to remove particulates and transient bacteria before we collected a skin swab for bacterial culture. For the bacterial cultures, all *B. luciae* were released within 10 min of capture, but the eight *A lugubris*, were first sampled for bacteria and then transported to the laboratory for use in the susceptibility trial. Each swab was suspended in a microfuge tube with 1 mL of DS solution, a salt solution resembling pond water (56) and transported to the laboratory for bacterial culturing.

A cell free supernatant (CFS) inhibition assay was used to determine whether cultured bacteria produced anti-fungal activity (57). The cultured *Bd*-GPL (strain id#CJB57-(4)-p6) used in the susceptibility trials was also used in this assay (17). Microfuge tubes containing DS solution and bacterial swabs from the field were vortexed within 24 h after field collection. Bacterial isolates in DS solution were incubated on R2A media and morphologically distinct bacteria were placed in axenic culture. *Bd* zoospore growth was then challenged against the various bacterial isolate supernatants (in replicates of five) in an adapted version of a previously published protocol (57). The optical density at 490 nm in each well was measured every 24 h using a SpectraMax 190 Microplate Reader and SoftMax Pro software. The total change in optical density after 7 days was used as a proxy for *Bd* growth. The average percent growth, normalized to the positive control, was calculated for each isolate using a previously published equation (58). Isolates that exhibited significantly lower % Growth values than the positive control were labeled as “Inhibitors” and isolates that did not differ significantly from the positive control were labeled as having “No Effect.”

In order to identify bacterial isolates, the 16S rDNA region of each isolate was sequenced at the San Francisco State University Genomics/Transcriptomics Analysis Core (GTAC) facility laboratory. Bacterial DNA was extracted using either Chelex or direct colony lifts into PCR reactions. The 16S rDNA region was amplified in PCR reactions using the 515F and 1492R 16S primers. The 16S regions were sequenced using standard protocol for chain termination sequencing, including an ExoSAPit PCR product purification, followed by cycle sequencing with BigDye 3.1 and the same 515F and 1492R primers as the initial PCR reaction.

Sequencher version 4.9 was used to assemble contigs from forward and reverse reads. Sequence data was then used to identify closest species match through BLAST, using default parameters for megablast searches. In order to confirm the genus of each isolate, sequence data was bootstrapped in MEGA version 6.06 against reference sequences belonging to the same family as the isolate's closest species match in BLAST. 16S rDNA reference sequences were selected from The NCBI Reference Sequence Database (RefSeq). Following a bootstrap sensitivity test, a tree was constructed for each isolate using the neighbor joining method and bootstrapped 500 times.

### Statistical Analyses

All statistical analyses were performed using the software R (version 3.5.0) in RStudio (version 1.1.447). All of the code used are freely available on Zenodo (https://zenodo.org/badge/latestdoi/137778706). To characterize the temporal distribution of *Bd* in *B. luciae* and *A. lugubris* within our historical data, we calculated 95% credible intervals for *Bd* prevalence in each decade sampled based on the binomial probability distribution, using sample size and the number of *Bd* positive individuals (28, 59). To test the likelihood of detecting a *Bd*-positive individual in each decade sampled, we calculated the probability of detecting zero positives using the binomial distribution. Two previous studies that used an identical qPCR assay found an enzootic level of ~11% prevalence in amphibian populations in Illinois, USA (60), and in another California slender salamander *Batrachoseps attenuatus* (21); therefore, we used 0.11 as the probability of detecting a *Bd* positive individual.

We performed a stepwise binomial logistic regression on field-collected individuals of both species using *Bd* infection status as the response variable with the R stats and MASS packages (version 3.5.0, version 7.3-50; respectively). We used the following variable as our explanatory variables: precipitation, minimum temperature, maximum temperature, mean temperature, snout-to-vent length (i.e., the size of the animal as measured from the nares to the cloaca), elevation (meters above sea level), the number of conspecific hosts found with the individual, and the number of heterospecific hosts found within a 15 m radius of the individual. Using the skin swab collection date of every individual host (January 2014–December 2015), we matched the month and year for the climate variables using monthly averaged data from the PRISM Climate Group, Oregon State University (http://prism.oregonstate.edu, created 25 May 2018). To validate the model, we performed k-fold cross validation, subsetting our data into 100-folds to minimize bias in producing the folds using the package DAAG [version 1.22; (61)]. Survival estimates for our laboratory infection trials were performed using the R package “survival” [version 2.42-3; (62)]. In the bacterial *Bd* inhibition trials, percent growth values of each isolate were compared to the positive control using a two-tailed Student's *t*-test, with significant *p*-value cutoffs (*p* < 0.005 for isolates from *A. lugubris* eggs [*n* = 9], *p* < 0.003 for isolates from *A. lugubris* [*n* = 16], and *p* < 0.003 for isolates from *B. luciae* [*n* = 15]) determined using the Bonferroni correction to minimize false-positives (63).

## Results

### Historical Infection Study (1920–2015)

**Figure 2:**
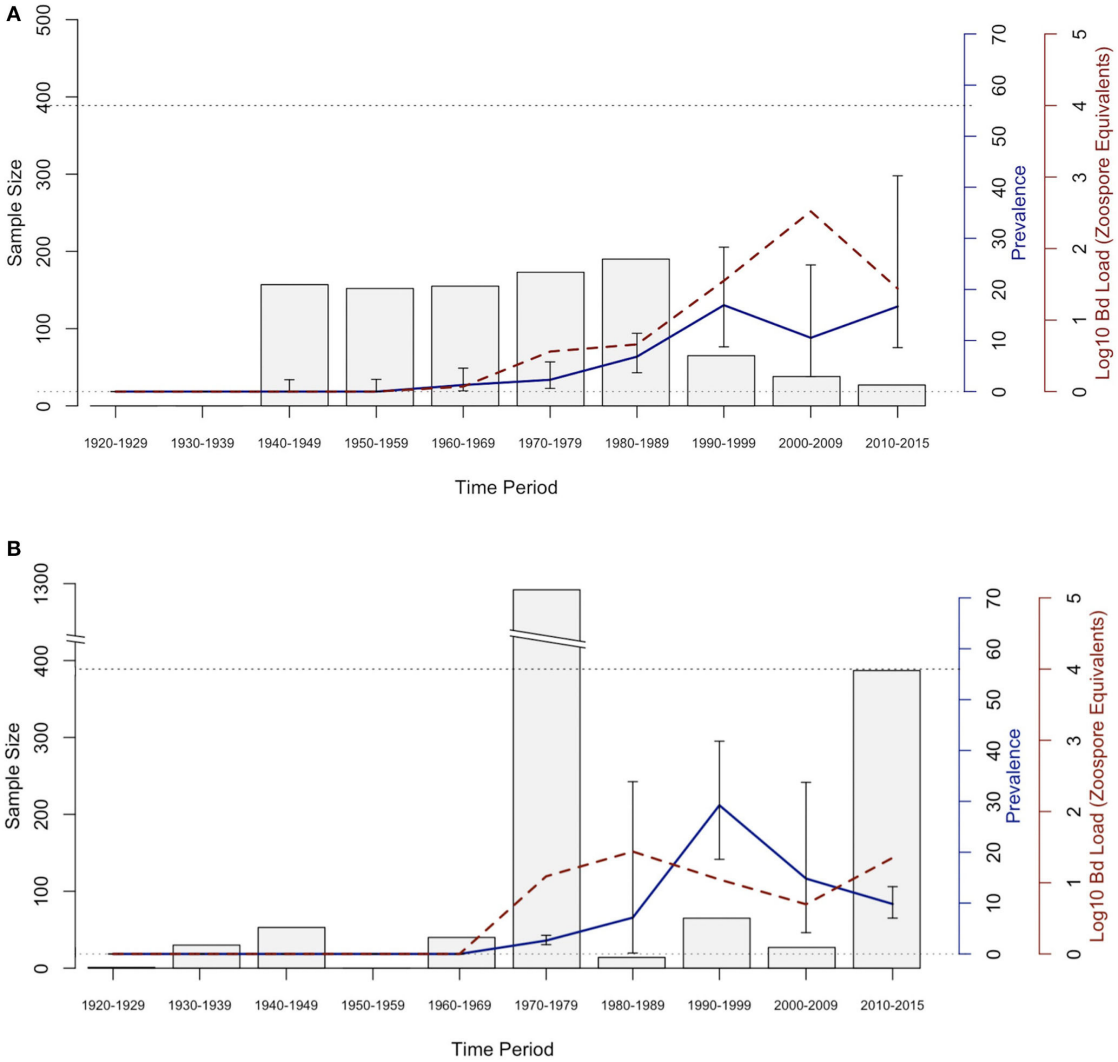
*Bd* prevalence and infection intensity by decade in museum specimens of **(A)** *Aneides lugubris* and **(B)** *Batrachoseps luciae*. From left to right, 1st Y-axis and bars represents sample size, 2nd Y-axis axis and blue line graph represents *Bd* infection prevalence, and the 3rd Y-axis and dotted red line represents *Bd* infection intensity (Log10 zoospore equivalents). Error bars represent credible intervals of *Bd* infection prevalence based on a binomial distribution and a 0.11 expected *Bd* prevalence from an area where *Bd* is assumed to be endemic (21, 60).

The historical samples spanned the entire range of *B. luciae* and nearly the entire range of *A. lugubris* (Figure 1). All *B. luciae* specimens in the decades of the 1920s−1960s (*n* = 123) were negative for *Bd* (Table 1). The first *Bd*-positive individuals of *B. luciae* were collected in 1972, and after that time, prevalence began to steadily increase (Figure 2A). *A. lugubris* was found to have a similar pattern; the first *Bd*-positive individual was from 1968, after more than 300 specimens tested negative (1940–1972; Table 1), and after that *Bd* prevalence increased (Figure 2B). For both species, *Bd* prevalence appears to trend higher in the 1980s and 1990s (Figures 2A,B). In *B. luciae, Bd* prevalence peaked at 29.23% in the 1990s and then subsided to 9.82% in 2010–2015. In *A. lugubris, Bd* prevalence increased to 16.92% in the 1990s and remained at 16.67% in 2010–2015. Both species show an upward trend in infection intensity through the 1990s, though few individuals surpassed Vredenburg's 10,000 Zoospore Rule, the expected lethal infection intensity for *Bd* in anurans (17, 54). When we compared the *Bd* prevalence from the decade with the highest prevalence (1990s for both species) in the museum specimens to the prevalence in contemporary field samples (collected 2014–2015; Table 2), we found that *Bd* prevalence had decreased significantly from 29.23 to 9.87% in *B. luciae* (*p* < 0.01; *X*^2^ = 18.8), but showed no significant difference in *A. lugubris* (16.92% compared to 18.18%; *p* = 0.87; *X*^2^ = 0.03).

**Table 1:** *Batrachochytrium dendrobatidis* (*Bd*) prevalence in the Santa Lucia Mountains slender salamander (*Batrachoseps luciae*) and the arboreal salamander (*Aneides lugubris*) museum specimens collected in California.

**Species**	**Decade**	**Sample size**	***Bd* positive**	**% Prevalence**	**95% CI**		
					***Lower***	***Upper***	***Pr (no Bd)***
*B. luciae*	1920–29	1	0	0	0	0	0.89
	1930–39	30	0	0	0	0	0.03
	1940–49	53	0	0	0	0	<0.01
	1950–59	0	0	0	0	0	1
	1960–69	40	0	0	0	0	<0.01
	1970–79	1,292	34	2.63	1.829	3.658	<0.01
	1980–89	14	1	7.14	0.181	33.87	0.19
	1990–99	65	19	29.23	18.6	41.82	<0.01
	2000–09	27	4	14.81	4.189	33.73	0.04
	2010–15	387	38	9.82	7.043	13.23	<0.01
	**Total**	**1,909**	**96**				
*A. lugubris*	1920–29	0	0	0	0	0	1
	1930–39	0	0	0	0	0	1
	1940–49	157	0	0	0	2.322	<0.01
	1950–59	152	0	0	0	2.397	<0.01
	1960–69	155	2	1.29	0.156	4.583	<0.01
	1970–79	173	4	2.31	0.633	5.814	<0.01
	1980–89	190	13	6.84	3.693	11.416	<0.01
	1990–99	65	11	16.92	8.762	28.266	<0.01
	2000–09	38	4	10.52	2.943	24.805	0.01
	2010–15	27	6	16.67	8.621	42.258	0.04
	**Total**	**957**	**40**				

**Table 2:** *Batrachoseps luciae* and *Aneides lugubris* field *Bd* prevalence by location, May 2014-March 2015.

**Species**	**Location**	**Sample size**	**Bd positive**	**% Prevalence**	**95% Credible Intervals**	
					***Lower***	***Upper***
*B. luciae*	Don Dahvee	188	19	10.11%	0.07	0.15
	Veterans Memorial	16	1	6.25%	0.01	0.29
	Van Winkle	137	15	10.94%	0.68	0.17
	Mission Trails	36	1	2.78%	0.01	0.14
	Other	8	2	25%	0.07	0.60
	**Total**	**385**	**38**	**9.87%**	–	–
*A. lugubris*	Monterey Co.	21	5	23.81%	0.11	0.45
	Other	12	1	8.33%	0.02	0.36
	**Total**	**33**	**6**	**18.18%**	–	–

### Contemporary Field Study and Generalized Linear Model

For *B. luciae* swabbed in the field between 2014 and 2015, 38 of 385 (9.87%) were *Bd* positive (Table 2); in contrast, 6 of 34 (18%) field-swabbed *A. lugubris* were *Bd* positive (Table 2). Of the 34 *A. lugubris* individuals sampled, 21 were located in Monterey County and 13 were located in San Mateo, Alameda and Calaveras counties. All georeferenced data (excluding the laboratory trial data) from the contemporary populations (catch and release animals) and from the museum specimens sampled are freely available for download at AmphibiaWeb's Amphibian Disease Portal (AmphibianDisease.org; https://amphibiandisease.org/projects/?id=251; doi's available at: https://n2t.net/ark:/21547/DTN2 - field based samples; https://n2t.net/ark:/21547/DoZ2 - museum based samples), a global archive for chytrid sampling data (see Koo et al., this issue).

The binary logistic regression model with the best (lowest) AIC (AIC = 225.4; Table 3), *Bd* infection status had a positive relationship with the following variables: elevation, number of heterospecifics, and number of conspecifics (*p* = 0.01, *p* < 0.01, *p* < 0.01; respectively). Precipitation had a negative relationship with *Bd* infection status (*p* = 0.01), and mean temperature had no significant relationship with *Bd* infection status (*p* = 0.09). Our k-fold cross-validation of the best model showed an estimate accuracy of 90% for the best model.

**Table 3:** Top 4 models of the stepwise binary logistic regression for *B. luciae* and *A. lugubris* with *Bd* infection status as the dependent variable.

**Variables**	**Model 1**	**Model 2**	**Model 3**	**Model 4**
Precipitation (–)	X	X	X	X
Mean temperature	X	X	X	X
Elevation (+)	X	X	X	X
Number of conspecifics (+)	X	X	X	X
Number of heterospecifics (+)	X	X	X	X
Species	X	X	X	
Maximum temperature	X	X		
Snout-to-vent Length	X			
AIC	229	228	226	225
ΔAIC	NA	1	2	1

*(+) Indicates a significant positive relationship with Bd infection status while (–) indicates a negative relationship with Bd infection status in the best model (model 4, AIC = 225)*.

In addition to revealing an effect of conspecifics in the binary logistic regression model (Table 3) we separated all field sampled individuals for *B. luciae* into their respective social group sizes, as determined by presence together under the same cover item. There was a clear pattern where the probability of infection increased 2x to 3x in social groups relative to individuals who were found to be solitary (Table 4). Interestingly, there was no effect of social group size (2 vs. 3 or 4+) on the prevalence of *Bd*, revealing that sociality itself rather than size of social group is the best indicator of *Bd* risk (Table 4).

**Table 4:** Effects of *B. luciae* group size on *Bd* infection prevalence: “Group size” = number individuals under cover item; “No. groups” = number of replicate groups found of each size; “No. individuals” = total individuals observed of each group size category; “No. sampled” = number of individuals observed that were swabbed; “No. infected” = number of sampled individuals that were *Bd* positive.

**Group size**	**No. groups**	**No. individuals**	**No. sampled**	**No. infected**	**% sampled infected**
1	164	164	164	8	4.9%
2	41	82	76	8	10.5%
3	17	51	50	7	14%
4 or more	20	148	95	15	15.8%

### Laboratory Host Susceptibility Trials

**Figure 3:**
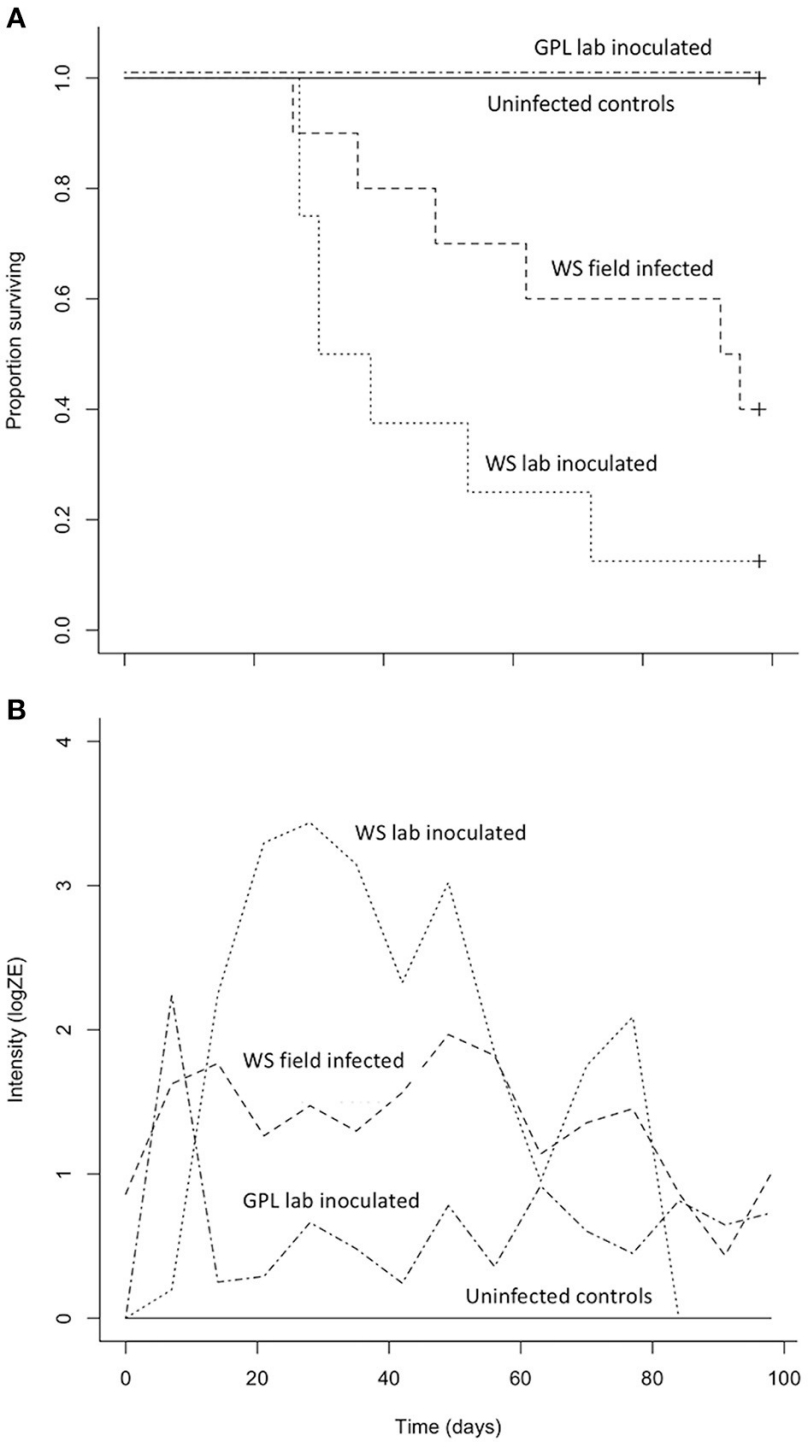
Results of *Batrachoseps luciae Bd* susceptibility trial [four treatment groups; wild-strain (WS) lab-inoculated (*n* = 8; dotted line), wild-strain (WS) field infected (*n* = 10; dashed line), GPL (global panzootic lineage) lab-inoculated (*n* = 8; solid line), and uninfected control group (*n* = 5; dash and dot line)] showing Kaplan-Meier survival estimate **(A)** illustrating proportion of individuals surviving over time, and **(B)** average *Bd* infection intensity (zoospore equivalents, log scale) over time.

**Figure 4:**
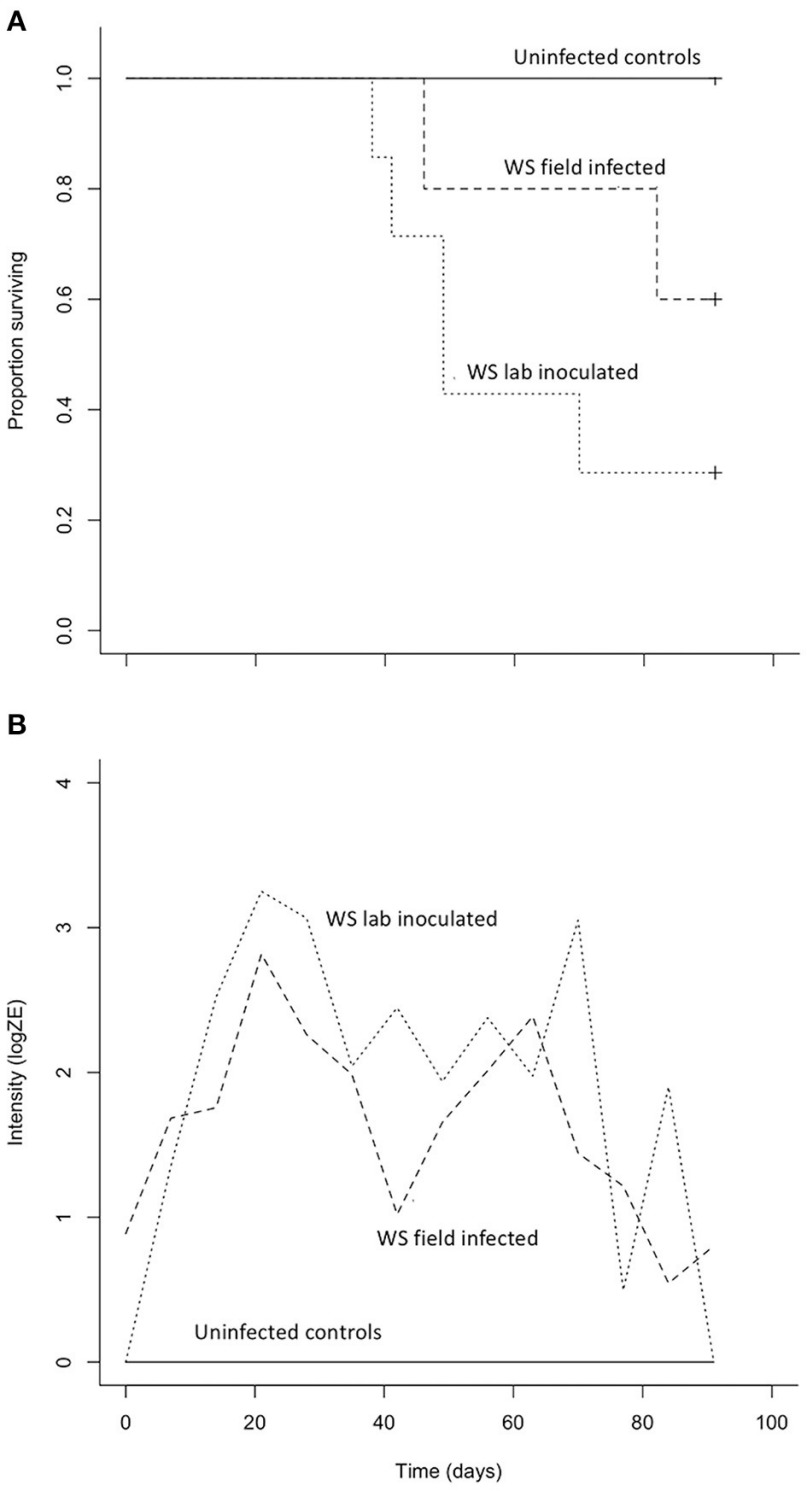
Results of *Aneides lugubris Bd* susceptibility trial [three treatment groups; wild-strain (WS) lab-inoculated (*n* = 7; dotted line), wild-strain (WS) field infected (*n* = 5; dashed line), and uninfected control group (*n* = 6; solid line)] with Kaplan-Meier survival estimate **(A)** illustrating proportion of individuals surviving over time, and **(B)** average *Bd* infection intensity (zoospore equivalents, log scale) over time.

Both *B. luciae* and *A. lugubris* suffered high mortality from chytridiomycosis in the laboratory experiments (Figures 3A, 4A), yet all of the uninfected controls for both species survived the entire experiment with no mortality and no positive *Bd* test results from the qPCR assay. None of the *B. luciae* exposed to *Bd*-GPL (GPL lab-inoculated; *Bd*-GPL, strain id#CJB57-(4)-p6) died (Figure 3A), however they did become infected (Figure 3B). High mortality was observed among *B. luciae* in the WS field-infected and WS lab-inoculated groups, with 60 and 87.5% mortality, respectively. When mortality between the two groups (WS field-infected and WS lab-inoculated) was compared, we found no significant difference between them (*p* = 0.08). However, there was a significant difference in mortality rates between WS field-infected and uninfected control groups (*p* < 0.05; Figure 3A), and between WS lab-inoculated and uninfected controls (*p* < 0.001; Figure 3A). In *A. lugubris*, we also found a significant difference in survival (*p* < 0.02; Figure 4A) between WS lab-inoculated *A. lugubris* (71.4% mortality) compared to the uninfected controls (Figure 4A). Although mortality was observed in WS field-infected individuals (40% mortality), there was no significant difference in mortality rates between the WS field-infected and uninfected control groups (*p* = 0.3; Figure 4A). When we compared the *Bd*-infection intensity of individuals in our different groups (Figures 3B, 4B; uninfected values not included), we consistently found higher levels of infection in groups that suffered mortality, but the differences, compared as averages compiled for each group, were not significantly different.

### Host Skin Bacteria *Bd* Inhibition Trials

A total of 32 bacterial samples were identified using 16S rDNA sequencing (Supplementary Table 1). The majority of bacterial isolated demonstrated significant *Bd* inhibitory activity against *Bd*-GPL, strain id#CJB57-(4)-p6. Thirteen isolates were identified from five *B. luciae* hosts' skin microbiome; and, all of the isolates demonstrated significant levels of *Bd* inhibition. The *A. lugubris* skin microbiome from three hosts yielded 13 isolates (Supplementary Table 1) and 10 of them demonstrated *Bd* inhibition. We also identified 6 bacterial isolates from the *A. lugubris* egg mass, and half of the isolates demonstrated similar *Bd* inhibitory activity. Isolates represented 4 bacterial phyla, Proteobacteria, Firmicutes, Actinobacteria, and Bacteroidetes. Isolates from *B. luciae* skin represented Proteobacteria (61.5%), Firmicutes (23.1%), and Actinobacteria (15.4%). *A. lugubris* skin bacterial isolates mirrored these phyla representations; Proteobacteria (76.9%) was the dominant phylum, followed by Firmicutes (15.4%) and Actinobacteria (7.7%). Isolates from *A. lugubris* eggs also had Proteobacteria (50%), Bacteroidetes (33.6%), and Firmicutes as the most abundant Phyla (16.7%). Inhibitory isolates were found to span all 4 phyla, with only Proteobacteria and Firmicutes containing isolates that had no effect on *Bd* growth.

## Discussion

The results from our 90-year retrospective museum study suggest that *Bd* emerged in *B. luciae* and *A. lugubris* in the late 1960s and early 1970s, with prevalence and infection intensity steadily increasing into the 1990s. In *B. luciae*, the proportion of *Bd*-infected specimens tested decreased from a high near 30% *Bd*-positive in the 1990s to below 10% *Bd*-positive in more recent time periods. A similar temporal pattern has been documented in previous retrospective studies of amphibians in California (19, 21, 22, 25, 28, 30, 32). Thus, our study provides further support for the hypothesis that *Bd* invaded California, but establishment, emergence and spread occurred several decades after *Bd* was first detected. This emergence is roughly coincident with two of the earliest documented mass-die off events for anurans (frogs) in North America, both in 1978 (32, 64, 65). In addition, we suggest that the temporal patterns of *Bd* that we describe may signal a shift from epizootic dynamics in the 1970–1990's to more stable enzootic dynamics in the years since 2000 (20, 45). This pattern is consistent with the one proposed for the Sierra Nevada yellow-legged frog, *Rana sierrae* (66), that suffered a major decline and appears to be recovering after nearly four decades despite *Bd* infections remaining in host populations (45). However, *Bd* infections may still slow population growth even in species that may have survived epizootics (20, 46).

We find no indication of a unidirectional spread of *Bd* across a large geographic range, which is consistent with previous studies on historical *Bd* invasion in California (19, 21, 25, 28, 30). Previous studies found evidence that *Bd* may have invaded California earlier than we detected in our study (67, 68), but as new evidence comes to light, it seems clear that those early invasions of *Bd* did not become established or spread widely until several (~4–5) decades later (19, 21, 22, 30). Our results are consistent with a regional pattern (western North America) showing a geographic expansion of collection localities for *Bd*-positive samples, as well as a large increase in the number of *Bd*-positive samples detected beginning ~1969–1972, and is consistent with previous work (19, 21, 25, 28, 30). What caused the invasion of *Bd* to California remains unclear, but the introduction of non-native amphibians is one likely factor (26, 32, 67, 68), and there are indications this also could be important globally (24, 69–71). The invasion of *Bd* in western North America and elsewhere was unnoticed because the first epizootics [e.g., frogs in the Sierra Nevada; (19)] occurred two or three decades before *Bd* was first discovered (10, 14). For cryptic, understudied species like terrestrial salamanders in California, we hypothesize that it is possible they experienced un-documented *Bd*-epizootics in the past [“ghost of epidemics past”; (23)], but also escaped notice due to their cryptic life history, behavior, and relatively unstudied populations compared to frogs [e.g., unlike salamanders, many frogs have large, often noticeable populations, schools of larvae-tadpoles, and vocalize during mating; (72)].

Our field study indicates that both *B. luciae* and *A. lugubris* populations in the Monterey Bay Area, California, currently sustain *Bd* infection prevalence at or slightly below 20% without visible epizootics (we found no carcasses and infected individuals appeared healthy when captured, only to die later in captivity from chytridiomycosis). Because both *B. luciae* and *A. lugubris* contract *Bd* in the wild and overlap in both diet and microhabitat, it is likely that interspecific contact plays a role in disease transmission. Our logistic regression found that the number of *A. lugubris* in proximity to *B. luciae* does, in fact, correlate positively with higher disease prevalence and infection intensity in *B. luciae*. The same is not true, however, when considering the proximity of *B. luciae* to *A. lugubris*, which may be a sample size issue because we did not find as many *A. lugubris*. It is interesting to note that *A. lugubris* have a much broader distribution, and have almost twice the *Bd* infection prevalence, which may suggest that *A. lububris* could be a source of *Bd* infection in *B. luciae* and perhaps other species. Nevertheless, the mechanism behind any asymmetric effect of co-infection remains unclear. One possibility is that *A. lugubris* is a more effective vector of *Bd* than *B. luciae;* for example*, A. lugubris* are much larger and as such, may shed more infective zoospores than a much smaller *B. luciae*. The shedding rate of *Bd* zoospores is thought to directly affect infection dynamics (53, 55, 73). It is possible that predation attempts by *A. lugubris* on *B. luciae* (rather than vice versa) may provide opportunities for individual contact between the two species or increase stress in the prey, which could facilitate the unidirectional transfer of zoospores or increased susceptibility of infection from predator to prey, increasing the transmission rate and ultimately the infection prevalence of the prey species*, B. luciae*.

Our results showing a positive relationship between sociality and *Bd* prevalence suggest that, in *B. luciae*, attraction to conspecifics is likely to facilitate disease spread. It is well-known that both the overall size of social groups as well as their intrinsic structure can facilitate parasite transmission (34, 35). A social structure involving high rates of behavioral interaction among hosts within a group can result in rapid and sustained pathogen spread, especially when individuals are susceptible to re-infection (74, 75). Individual *B. luciae* in social groups were often found in direct skin-to-skin contact, similar to other species studied within the same genus (21, 25). Under these conditions of frequent contact, social organisms are known to experience higher rates of infection and pathogen spread in a population (R^0^), even when infection arises from a single initially infected individual (76, 77). Despite the costs of group living that relate to parasites and pathogens, it has also been shown that social behavior can benefit hosts by spreading beneficial microbes important in disease resistance (78, 79). While our study found a behavioral effect, with larger groups of *B. luciae* having higher infection prevalence, it is possible that these social groups also harbored more protective microbes, which could mitigate the effect of the fungal pathogen [as we found in our skin bacterial cultures; (80)]. However, there are several alternative explanations. For example, *Bd*-infected *B. luciae* may prefer to group, while uninfected individuals prefer to be solitary, or there could be some environmental factors, not included in this study, that could make some locations more favorable for the hosts and lead to host aggregation. A retrospective study in *Batrachoseps attenuatus*, a closely related and similar species, found a *Bd* infection pattern consistent with our study: *Bd* infection was positively associated with host group size (21). This study also revealed that host populations with longer *Bd* exposure histories (over several decades) were significantly less social (i.e., had smaller average group sizes) than host populations with either a short history of infection (months to a few years) or populations very recently infected by *Bd* (21). These combined results suggest that sociality may increase *Bd* transmission rate while also leading populations with long-term exposure to evolve away from the proposed ancestral mode of sociality.

When uninfected wild collected *B. luciae* and *A. lugubris* were exposed to wild strain *Bd* (WS lab-inoculated) they were highly susceptible and experienced high mortality, ~90%, and >70%, respectively. In both species, individuals that succumbed to the disease exhibited similarly high levels of *Bd* infection intensity consistent with signs of chytridiomycosis as described by the Vredenburg 10,000 Zoospore Rule (17, 54). Interestingly, *B. luciae* were not susceptible when exposed to *Bd*-GPL. This *Bd* genotype has been sequenced and described as the “Global Panzootic Lineage” [*Bd*-GPL (39)], and is the same lineage found in *Bd* epizootics that were responsible for species extinction events in Central and South America and Australia (12, 23). Different genotypes of *Bd* have varying levels of virulence on hosts (12, 24, 38, 81). Of the most widespread and well-known *Bd* lineages, *Bd*-GPL is the most virulent on adult hosts (38). The fact that we found no mortality of *B. luciae* when exposed to *Bd-*GPL may indicate that hosts are adapted to this lineage, or that the culture had decreased in virulence in captivity. Other studies have proposed similar ideas regarding host adaptation to *Bd* lineages [(40); reviewed in (12)]. The cultured *Bd*-GPL we used to expose *B. luciae* was collected from *Rana muscosa* populations experiencing *Bd* epizootics that subsequently drove populations to extinction (17). For this study, the *Bd*-GPL culture was revived from cryopreserved culture before the trials; we did not use a culture that was continually kept active. Cryopreservation decreases passage rates, which are known to decrease virulence in many pathogens kept cultured in laboratory conditions (82). The *Bd-*GPL culture used here was lethal to frogs in other *Bd* laboratory susceptibility experiments using the exact same exposure protocol we used (45, 50, 53). Along with previous studies, we conclude that pathogen lineage should be carefully considered when designing host susceptibility trials and should be linked with contemporary field studies whenever possible (83, 84).

For this study, we also inoculated individuals from both salamander species with *Bd* from live, collected wild *Bd*-infected salamanders. Laboratory conditions vary greatly from the wild, and we suggest that laboratory susceptibility trials are best interpreted in conjunction with other data (e.g., spatio-temporal infection prevalence, host behavior, and other biotic and abiotic factors) that could help describe the particular context of the host/pathogen dynamics. Despite extensive searches, we did not find sick or dying animals in our field surveys, and population declines have not been reported for these two species; however, there are also no population surveys available that could be used to detect declines for these species. In addition, the absence of sick or dying individuals in the field alone is not enough evidence to suggest that the pathogen is not affecting populations in the wild (23, 46). Salamanders are known to quickly decompose in the wild after death, and thus ascertainment bias may occur. This is especially important to consider given the fact that the wild strain *Bd* caused high mortality in both *B. luciae* and *A. lugubris* individuals in our laboratory susceptibility trials (Figures 3A, 4A). In fact, to our knowledge, most population extinctions caused by *Bd* epizootics revealed few sick animals or carcasses [but see (17, 85)] before populations disappeared, perhaps because amphibian carcasses are rapidly consumed by predators, scavengers, and saprophytic microbes. Finally, it is important to recognize that for terrestrial salamanders, dead individuals are not expected to be conspicuous since these species live under cover items and/or underneath the soil surface. Unlike previous studies mainly on frogs where hosts are exposed to *Bd* while in aquatic habitats, our host species are exposed to *Bd* in terrestrial habitats and never go to aquatic habitats. How this impacts chytridiomycosis dynamics is poorly understood.

There is a growing body of evidence that host skin microbiome communities affect host health. Several studies have shown that *Bd-*inhibiting skin bacteria from hosts can inhibit *Bd* growth and limit the effects of *Bd* on susceptible amphibians (36, 80, 86, 87), and *Bd* inhibiting skin bacteria occur on many amphibian species (58, 88, 89), including terrestrial salamanders that live in California (90, 91). In this study we found *Bd* inhibiting bacteria on both focal salamander species, with a high percentage of culturable species exhibiting *Bd* inhibition (*B. luciae*, 100%; *A. lugubris*, 81.2%, and *A. lugubris* eggs, 75%) compared to other studies (58, 89, 92, 93). The swab culturing methodology used in this and other studies, however, is not perfect. For example, we did not test for other organisms (e.g., fungi) in the swab cultures, and it is possible that they could affect our bacterial culturing efforts. The high percentage of *B. luciae* bacterial isolates that inhibited the *Bd*-GPL culture is in accordance with our susceptibility trial data, in which *B. luciae* exposed to this *Bd* culture exhibited no mortality. This suggests that *Bd* inhibitory isolates on *B. luciae* skin may help this salamander species resist *Bd* induced mortality. The wild *Bd* was not available in culture, and thus we cannot draw direct conclusions about the bacterial inhibition on it. However, since previous studies examining populations of threatened amphibians have revealed a correlation between presence of *Bd* inhibitory symbiotic skin bacteria and resistance to *Bd* infections in the wild (87, 94, 95), we propose that some of the skin bacteria found living on *B. luciae* and *A. lugubris* may provide a mechanism for survival of *Bd*-infected individuals in the wild.

Our retrospective analysis shows *Bd* invasion, establishment, and emergence in two sympatric species of terrestrial salamanders. This is consistent with patterns in other hosts from previous retrospective studies of *Bd* in California (19, 21, 22, 25, 28, 30, 32). We suggest that other amphibian species in California, including the most diverse group [salamanders; (1)], may have been negatively affected by this pathogen in the past, but because the invasion and emergence occurred before the pathogen was described, this phenomenon may have been overlooked [“ghost of epizootics past”; (23)]. Population monitoring of salamanders that live cryptically is challenging, which makes it likely that any present or past effects of *Bd* on salamander populations may not have been measured. Although the two species we studied differ greatly in range size, abundance, and social behavior, the timing of emergence in both species is strikingly similar to each other and to other studies in western North America (19, 21, 30). However, our results are in stark contrast to studies that used the exact same techniques to test for *Bd*-infection in museum specimens collected in other areas. For example, Talley et al. (60) found *Bd*-infected specimens consistently in ~11% of specimens dating across over a century of collections (as far back as the 1890s) in specimens collected throughout Illinois, USA. In Brazil, Rodriguez et al. (96) found *Bd*-infected specimens consistently in ~40% of specimens dating across all decades going back 100 years. For both of these studies, many *Bd*-infected specimens were discovered even in the oldest specimens, suggesting that the technique developed by Cheng et al. (50) is robust.

We found that heterospecific hosts may influence disease. For example, the presence of *A. lugubris* may influence disease prevalence and infection intensity of *B. luciae*. We found that *A. lugubris* have high prevalence of *Bd* in wild populations, which could possibly increase the chances of *Bd* transmission to other heterospecific hosts. Other species have been proposed as *Bd* reservoirs (53) or *Bd* super shedders (97), and *A. lugubris* shares some similar qualities. The arboreal salamander, *A. lugubris*, has an expansive geographic range that overlaps in distribution with 28 of California's 49 salamander species (1), has a relatively high proportion of infected individuals in nature, and is able to infect other host species (this study). This species is often found in close proximity to other hosts (terrestrial salamanders genera: *Aneides, Batrachoseps, Ensatina*), even under the same cover items (e.g., woody debris such as logs, bark). Several amphibian species sympatric with *A. lugubris* have been identified as *Bd-*positive (21, 22, 25, 30), but little is known about their susceptibility to *Bd* or whether their infection status might also be influenced by proximity to *A. lugubris* individuals. Reservoir species, and super shedders also pose a risk in that they could spread novel pathogens that may invade in the future (26). For example, *Batrachochytrium salamandrivorans* (*Bsal*), another recently discovered chytridiomycete pathogen, has caused mass die offs in wild salamander populations in Europe (98–101), but has not been found in North America (102). *Bsal*-infected animals are present in the international amphibian pet trade (103, 104), and this puts North American salamanders, including the terrestrial salamanders, at further risk of new epizootic disease (26, 105, 106). If we hope to use disease theory to help predict and mitigate disease risk in amphibians, we suggest that additional studies on chytridiomycosis in terrestrial salamanders are urgently needed.

## Data Availability Statement

The datasets presented in this study can be found in online repositories. The names of the repository/repositories and accession number(s) can be found at: https://amphibiandisease.org/projects/?id=251, https://n2t.net/ark:/21547/Ajp2.

## Ethics Statement

The animal study was reviewed and approved by San Francisco State University Institutional Animal Care and Use Committee.

## Author Contributions

MC, VV, and AZ conceived and designed the experiments. MC and WS performed the experiments and conducted fieldwork. MC, HS, WS, TY, VV, and AZ analyzed the data. MC, WS, TY, HS, VV, and AZ wrote the manuscript. MK provided editorial advice. All authors contributed to the article and approved the submitted version.

## Funding

This project was funded by NSF IOS – 1258133 AZ and VV, and through the Belmont Forum project: People, Pollution, and Pathogens (P3); NSF 1633948 to VV. Funding also came from San Francisco State University Instructionally Related Activities Grants awarded to MC, and from the Whitman Internship Grant awarded to WS. Publication made possible in part by support from the Berkeley Research Impact Initiative (BRII) sponsored by the UC Berkeley Library.

## Conflict of Interest

The authors declare that the research was conducted in the absence of any commercial or financial relationships that could be construed as a potential conflict of interest.

## Publisher's Note

All claims expressed in this article are solely those of the authors and do not necessarily represent those of their affiliated organizations, or those of the publisher, the editors and the reviewers. Any product that may be evaluated in this article, or claim that may be made by its manufacturer, is not guaranteed or endorsed by the publisher.
