# Supplementary material for: Social Behavior, Community Composition, Pathogen Strain, and Host Symbionts Influence Fungal Disease Dynamics in Salamanders
Source: Front Vet Sci. 2021 Nov 29;8:742288. doi: 10.3389/fvets.2021.742288 (PMC8687744; doi:10.3389/fvets.2021.742288)
Supplement: Supplementary file 1 [file Table_1.docx]

Supplemental (Appendix)

**Table S1.** Bacterial isolates were collected from adult *B. luciae* individuals, *A. lugubris* individuals, and *A. lugubris* eggs. The 16s rDNA gene of each isolate was sequenced and identified using BLAST and Neighbor-Joining tree construction/bootstrapping. Identity Scores for all BLAST hits are greater than 98% except when indicated with an *. % Growth normalized to the positive control was estimated and each p-value was calculated using a Two-Tailed Student’s T-test in R. Significance of each isolate’s inhibition of *Bd* was determined using the Bonferroni correction to establish a conservative p-value cutoff for significance (n=15, p < 0.0001).

| **Host** | | | **Species** | | **Genus (bootstrap score)** | | | **Normalized % Growth** | | **p-value** | **Activity** |
| --- | --- | --- | --- | --- | --- | --- | --- | --- | --- | --- | --- |
| *B. luciae* | [Bacillus sp. mixed culture J1-38 (KR029176.1)](http://blast.ncbi.nlm.nih.gov/Blast.cgi#alnHdr_930010506) | | | | | Bacillus (100) | | | -0.05% | 2.51E-03 | Inhibitor |
| *B. luciae* | Bacillus sp. Ja24 (KR088402.1) | | | | | Bacillus (86) | | | -0.01% | 2.53E-03 | Inhibitor |
| *B. luciae* | [Streptomyces sp. SM18 (KT875346.1)](http://blast.ncbi.nlm.nih.gov/Blast.cgi#alnHdr_938244892)* | | | | | Streptacidiphilus /Kitasatospora(87) | | | -0.01% | 2.53E-03 | Inhibitor |
| *B. luciae* | Achromobacter piechaudii strain NRC2 (KP145014.1) | | | | | Achromobacter (97) | | | 0.74% | 2.59E-03 | Inhibitor |
| *B. luciae* | [Achromobacter piechaudii strain NRC2 (KP145014.1)](http://blast.ncbi.nlm.nih.gov/Blast.cgi#alnHdr_793973385) | | | | | Achromobacter (95) | | | 0.02% | 2.53E-03 | Inhibitor |
| *B. luciae* | [Burkholderia megapolitana strain A3 (NR_042594.1)](http://blast.ncbi.nlm.nih.gov/Blast.cgi#alnHdr_343202308) | | | | | Burkholderia (100) | | | 0.30% | 2.52E-03 | Inhibitor |
| *B. luciae* | [Bacillus subtilis strain CICC10074 (KP877509.1)](http://blast.ncbi.nlm.nih.gov/Blast.cgi#alnHdr_58615438) | | | | | Bacillus (97) | | | 0.38% | 2.55E-03 | Inhibitor |
| *B. luciae* | [Achromobacter xylosoxidans strain ZSB6 (KR703650.1)](http://blast.ncbi.nlm.nih.gov/Blast.cgi#alnHdr_827343545) | | | | | Achromobacter (87) | | | 0.68% | 2.58E-03 | Inhibitor |
| *B. luciae* | Pseudomonas protegens strain IR56 (KJ396833.1) | | | | | Pseudomonas (84) | | | 2.72% | 2.76E-03 | Inhibitor |
| *B. luciae* | [Pseudomonas sp. mixed culture J6-51 (KR029254.1)](http://blast.ncbi.nlm.nih.gov/Blast.cgi#alnHdr_930010662) | | | | | Pseudomonas (82) | | | 4.93% | 2.52E-03 | Inhibitor |
| *A. lugubris* | | [Pseudomonas koreensis strain BK-9 16S (KJ812045.1)](http://blast.ncbi.nlm.nih.gov/Blast.cgi#alnHdr_673523891) | | | Pseudomonas (82) | | | -0.08% | | 2.51E-03 | Inhibitor |
| *A. lugubris* | | [Uncultured Pseudomonas sp. clone JXS2-52 (JN873217.1)](http://blast.ncbi.nlm.nih.gov/Blast.cgi#alnHdr_359472442)* | | | Pseudomonas (96) | | | -0.04% | | 2.52E-03 | Inhibitor |
| *A. lugubris* | | [Pseudomonas koreensis strain BK-9 (KJ812045.1)](http://blast.ncbi.nlm.nih.gov/Blast.cgi#alnHdr_673523891) | | | Pseudomonas (80) | | | -0.03% | | 2.52E-03 | Inhibitor |
| *A. lugubris* | | [Microbacterium sp. C8(2015) (KT767758.1)](http://blast.ncbi.nlm.nih.gov/Blast.cgi#alnHdr_929985406) | | | Microbacterium (100) | | | -0.01% | | 2.53E-03 | Inhibitor |
| *A. lugubris* | | [Pseudomonas sp. Iso RS1 (KP306738.1)](http://blast.ncbi.nlm.nih.gov/Blast.cgi#alnHdr_816848823) | | | Pseudomonas (84) | | | 0.00% | | 2.53E-03 | Inhibitor |
| *A. lugubris* | | [Uncultured Bosea sp. clone BC0153 (KC166814.1)](http://blast.ncbi.nlm.nih.gov/Blast.cgi#alnHdr_523453929) | | | Bosea (100) | | | 0.02% | | 2.52E-03 | Inhibitor |
| *A. lugubris* | | [Caulobacter sp. SS14.14 (KC160785.1)](http://blast.ncbi.nlm.nih.gov/Blast.cgi#alnHdr_441089382) | | | Caulobacter (96) | | | 0.02% | | 2.53E-03 | Inhibitor |
| *A. lugubris* | | Listeria seeligeri strain cicc 21671 (KP326349.1) | | | Listeria (92) | | | 0.03% | | 2.53E-03 | Inhibitor |
| *A. lugubris* | | [Acidovorax anthurii strain XD-3 (KP641171.1)](http://blast.ncbi.nlm.nih.gov/Blast.cgi#alnHdr_763866855) | | | Acidovorax (92) | | | 0.28% | | 2.55E-03 | Inhibitor |
| *A. lugubris* | | [Caulobacter sp. BE3-LECU2A-W (KF596691.1)](http://blast.ncbi.nlm.nih.gov/Blast.cgi#alnHdr_558520693) | | | Caulobacter (100) | | | 95.42% | | 8.06E-01 | No Effect |
| *A. lugubris* | | Staphylococcus sp. ECSMB11 (KJ425237.1) | | | Staphylococcus (85) | | | 99.68% | | 9.87E-01 | No Effect |
| *A. lugubris* (eggs) | | | [Uncultured Dyella sp. clone: i133 (AB974302.1)](http://blast.ncbi.nlm.nih.gov/Blast.cgi#alnHdr_891166554)* | Dyella (100) | | | -0.17% | | | 2.51E-03 | Inhibitor |
| *A. lugubris* (eggs) | | | [Chitinophaga sp. S6C1 (KJ923804.1)](http://blast.ncbi.nlm.nih.gov/Blast.cgi#alnHdr_682237262) | Chitinophaga (91) | | | 1.57% | | | 2.63E-03 | Inhibitor |
| *A. lugubris* (eggs) | | | [Stenotrophomonas maltophilia strain P4 (KF177140.1)](http://blast.ncbi.nlm.nih.gov/Blast.cgi#alnHdr_516334853)* | Stenotrophomonas (100) | | | 20.19% | | | 4.64E-03 | No Effect |
| *A. lugubris* (eggs) | | | [Staphylococcus sp. F157 (KT361117.1)](http://blast.ncbi.nlm.nih.gov/Blast.cgi#alnHdr_932253248) | Staphylococcus (98) | | | 32.66% | | | 6.99E-03 | No Effect |

FIGURE S1 (UNDER DEVELOPMENT)
